# Audit of appropriate consideration of anti-craving medication following alcohol detoxification in a north east addictions service

**DOI:** 10.1192/bjo.2021.936

**Published:** 2021-06-18

**Authors:** Joseph Thorne, Sophie Quarshie

**Affiliations:** Cumbria, Northumberland, Tyne And Wear NHS Foundation Trust

## Abstract

**Aims:**

NICE guidelines recommend that all patients who undergo a successful alcohol detoxification programme should be considered for treatment with acamprosate or oral naltrexone. This audit studied the proportion of patients considered for acamprosate or naltrexone treatment in a North-East Addictions Service.

- Primary aim
- To explore whether naltrexone/acamprosate had been considered for each patient completing alcohol detoxification.

Secondary aims

- what proportion of those offered agreed to be prescribed acamprosate/naltrexone
- whether these patients were being adequately followed up in terms of prescription

**Background:**

There is a significant evidence base for both naltrexone and acamprosate in the maintenance of abstinence in patients with alcohol addiction. NICE recommends the consideration of both medications for patients following successful alcohol detoxification from alcohol. The addictions service at Plummer Court in Newcastle upon Tyne has a comprehensive pathway for alcohol detoxification patients, which involves multiple reviews by keyworkers and medics. The attendance at these appointments is often poor, and it is often unclear whether these patients have been offered anti-craving medication.

**Method:**

A list of patients referred for inpatient or outpatient alcohol detoxification between June to August 2018 (n = 23) was curated. The progress notes were reviewed for any evidence that there had been clinical consideration of acamprosate/naltrexone. If evidence was found that the discussion had taken place, the notes were further scrutinised to assess if the client had accepted a prescription. The clinical documentation was further reviewed to see if follow-up for anti-craving medication was in place.

**Result:**

There was evidence that anti-craving medication had been considered in 47% of patients during the treatment process

In all but one case, acamprosate was offered rather than naltrexone

In cases where medication was offered, it was accepted in all but one case

Anti-craving medication was universally well tolerated

There was considerable difficulty with assessing who was following up the prescription. On scrutiny of the notes, several GPs had contacted addictions services stating that they would not prescribe acamprosate because of local policy prohibiting its prescription from Primary Care (this policy is in fact no longer current)

**Conclusion:**

Practice changed to offer patients monthly follow-up with addictions services for six months

Template letter sent out to GPs with discharge from addictions requesting acamprosate prescription, outlining current policy and offering support if GPs not comfortable

Audit presented to medical team. Treatment pathway amended to specify medical team's role in offering anti-craving medication at initial appointment

Re-audit in six months

